# A study of the wave dynamics of the space–time fractional nonlinear evolution equations of beta derivative using the improved Bernoulli sub-equation function approach

**DOI:** 10.1038/s41598-023-45423-6

**Published:** 2023-11-22

**Authors:** Anamika Podder, Mohammad Asif Arefin, M. Ali Akbar, M. Hafiz Uddin

**Affiliations:** 1 Department of Mathematics, Jashore University of Science and Technology, Jashore, 7408 Bangladesh; 2 Department of Applied Mathematics, University of Rajshahi, Rajshahi, 6205 Bangladesh

**Keywords:** Applied mathematics, Theory and computation, Engineering, Mathematics and computing

## Abstract

The space–time fractional nonlinear Klein-Gordon and modified regularized long-wave equations explain the dynamics of spinless ions and relativistic electrons in atom theory, long-wave dynamics in the ocean, like tsunamis and tidal waves, shallow water waves in coastal sea areas, and also modeling several nonlinear optical phenomena. In this study, the improved Bernoulli sub-equation function method has been used to generate some new and more universal closed-form traveling wave solutions of those equations in the sense of beta-derivative. Using the fractional complex wave transformation, the equations are converted into nonlinear differential equations. The achieved outcomes are further inclusive of successfully dealing with the aforementioned models. Some projecting solitons waveforms, including, kink, singular soliton, bell shape, anti-bell shape, and other types of solutions are displayed through a three-dimensional plotline, a plot of contour, and a 2D plot for definite parametric values. It is significant to note that all obtained solutions are verified as accurate by substituting the original equation in each case using the computational software, Maple. Additionally, the results have been compared with other existing results in the literature to show their uniqueness. The proposed technique is effective, computationally attractive, and trustworthy to establish more generalized wave solutions.

## Introduction

Fractional calculus (FC) has applications in diverse and widespread fields of science such as electromagnetics, biological population models, solid-state physics, optics, chemical kinematics, earthquake simulation, rocket motion, plasma physics, nuclear explosion, control theory, signals processing, fluid flow and other areas^1–3^. It is still a powerful tool for understanding complex systems, particularly in engineering and physical sciences. It is a more advanced version of the classical order integration and differentiation. It is interesting that explicit traveling wave solutions for non-linear fractional partial differential equations (NLFPDEs) are applicable in the contemporary context. Any real-world problem may be explained in terms of its physical importance using analytical solutions to NLFPDEs. Researchers have increasingly concentrated on analytical and numerical solutions of nonlinear partial differential equations, including integer and fractional orders, because software-based symbolic instruments like Maple, MATLAB, or Mathematica have quickly advanced in computer science. In recent generations, various analytical and semi-analytical techniques, such as the new generalized $(G^{\prime} /G)$-expansion approach^4^, the extended tanh-function method^5^, the Hirota’s bilinear method^6, 7^, the Riccati-Bernoulli sub-ODE method^8^, the new extended direct algebraic method^9, 10^, the modified direct algebraic method^11, 12^, the extended sinh-Gordon equation expansion method^13–17^, the improved Bernoulli sub-equation function method^18–20^, the extended Fan sub-equation method^21^, and the generalized exponential rational function method^22–24^ have been studied, and also employed for finding the new exact solutions of the famous NLFPDEs that developed in applied sciences. The improved Bernoulli sub-equation function (IBSEF) method is a straightforward, substantial, and sophisticated algebraic approach for finding reliable and trustworthy solutions to NLFPDEs. This IBSEF approach was first originated from the Bernoulli sub-equation function method (BSEFM)^25^.

The specified space–time fractional nonlinear Klein-Gordon (NLKG) equation and space–time fractional modified regularized long-wave (mRLW) equation are important models in physics and engineering. Those equations play a substantial role in mathematical physics and have many scientific applications such as solid-state physics, nonlinear optics, quantum field theory, shallow water waves, and plasma waves. The spinless ion is correctly described by the space–time fractional NLKG equation, which also characterizes the relativistic electrons in atom theory^26^. The space–time fractional mRLW equation is employed in oceanography to understand long-wave occupancy dynamics in the ocean, including tsunamis and tidal waves, which are essential for coastal hazard assessment and maritime safety^27^. It is also used in coastal and ocean engineering to model the propagation of water waves in shallow water, accounting for dispersion and dissipation effects. This is how we consider these two models for solving in this research to explain above mentioned phenomena properly. The space–time fractional NLKG equation has been examined using a number of different techniques such as Ege and Misirli^28^ solved this equation using the modified Kudryashov method related to Jumarie's modified Riemann- Liouville derivative. Sadiya et al.^29^ assessed this equation by the extended tanh-function method with conformable derivative and the $(G^{\prime}/G,1/G)$-expansion method was used to interpret this equation by Yasar and Giresunlu^30^. The $(G^{\prime}/G)$ and $(w/g)$—expansion^31^ approaches, the Riccati expansion^32^ method, are also applied to find the exact solutions of the space–time fractional NLKG equation. On the other hand, the space–time fractional mRLW equation was solved by the Ansatz method with Jumarie’s derivative developed by Guner and Bekir^33^. Uddin et al.^34^ studied this equation based on the exp-function and double $({G}^{\prime}/G,1/G)$ method with conformable fractional derivative. Kaplan et al.^35^ found the solution of this equation using the modified simple equation method with the modified Riemann–Liouville derivative. It is notable to observe that the stated models have not yet been examined by the IBSEF technique. As a consequence, the objective of this study is to improve the precision of possible solitons solutions to the space–time fractional NLKG and mRLW equations utilizing the IBSEF technique with beta-derivative. The contour, 3D, and 2D plots are used to describe the graphical representations of the solutions that were found with specific values for the free parameters. The proposed method is effective in constructing a variety of soliton solutions, quicker for simulating, and flexible.

The rest of this paper is scheduled as: The Atangana beta-derivative is introduced in section “Atangana beta-derivative”, and in section “Outline of the IBSEF method”, the IBSEF method has been described. In section “Analysis of closed form solutions”, the wave solutions for the space–time fractional NLKG equation and space–time fractional mRLW equation are outlined and physical explanations & graphical descriptions are briefly mentioned in section “Results discussion and physical explanation”. In section “Results comparison”, a comparison is made between the obtained solutions and the existing solutions. Finally, the conclusion is presented in section “Conclusion”.

## Atangana beta-derivative

For modeling intricate systems and processes, fractional derivatives offer a more flexible and precise tool. Classical integer-order derivatives are unable to effectively capture the non-integer-order behavior of many real-world phenomena. Such systems can be more effectively represented using fractional calculus. The definition of a fractional derivative, which has been studied on by several academics, includes the Jumarie-modified Riemann–Liouville, conformable, and Caputo fractional derivative^36–38^. The beta-derivative is a newly proposed fractional derivative introduced by Atangana et al.^39^ as follows:

1 $${\mathfrak{T}}^{\sigma }\left(f\left({\mathcalligra{t}}\right)\right)={\frac{{d}^{\sigma }(f\left({\mathcalligra{t}}\right))}{d{\mathcalligra{t}}^{\sigma }}}=\lim\limits_{\varepsilon \to \infty }{\frac{f\left({\mathcalligra{t}}+\varepsilon {\left({\mathcalligra{t}}+{\frac{1}{\Gamma \left(\sigma \right)}}\right)}^{1-\sigma }\right)-f({\mathcalligra{t}})}{\varepsilon }};0<\sigma \le 1.$$

The abovementioned definition (1) satisfies the following characteristics:

2 $${\mathfrak{T}}^{\sigma }\left({{a}}_{1} f\left(\mathcalligra{t}\right)+{{a}}_{2} g(\mathcalligra{t})\right)={{a}}_{1} {\mathfrak{T}}^{\sigma }\left(f\left(\mathcalligra{t}\right)\right)+{{a}}_{2} {\mathfrak{T}}^{\sigma }\left(g\left(\mathcalligra{t}\right)\right),\quad for\,\, all {{a}}_{1},{{a}}_{2} \epsilon {\mathbb{R}},$$

3 $${\mathfrak{T}}^{\sigma }\left(f\left(\mathcalligra{t}\right) g(\mathcalligra{t})\right)=g\left(\mathcalligra{t}\right){\mathfrak{T}}^{\sigma }\left(f\left(\mathcalligra{t}\right)\right)+f\left(\mathcalligra{t}\right) {\mathfrak{T}}^{\sigma }\left(g\left(\mathcalligra{t}\right)\right),$$

4 $${\mathfrak{T}}^{\sigma }\left(\frac{f\left(\mathcalligra{t}\right)}{g\left(\mathcalligra{t}\right)}\right)=\frac{g\left(\mathcalligra{t}\right){\mathfrak{T}}^{\sigma }\left(f\left(\mathcalligra{t}\right)\right)-f\left(\mathcalligra{t}\right) {\mathfrak{T}}^{\sigma }\left(g\left(\mathcalligra{t}\right)\right)}{g{(\mathcalligra{t})}^{2}},$$

5 $${\mathfrak{T}}^{\sigma }\left(f\left(\mathcalligra{t}\right)\right)={\left(\mathcalligra{t}+\frac{1}{\Gamma \left(\sigma \right)}\right)}^{1-\sigma }\frac{df(\mathcalligra{t})}{d\mathcalligra{t}},$$

6 $${\mathfrak{T}}^{\sigma }\left((fog)\left(\mathcalligra{t}\right)\right)={\left(\mathcalligra{t}+\frac{1}{\Gamma \left(\sigma \right)}\right)}^{1-\sigma }{g}^{\prime}\left(\mathcalligra{t}\right) {f}^{\prime}\left(g\left(\mathcalligra{t}\right)\right),$$

7 $${ }{\mathfrak{T}}^{\sigma } \left( {\mathfrak{c}} \right) = 0.$$

Based on previously mentioned features, the beta-derivative can easily convert the NLFPDEs into an ordinary differential equation that is non-linear with an integer order. Many researchers use the beta-derivative in various physical applications due to its flexibility, well-posedness of mathematical properties, and ability to generalize classical derivative to fractional order making it an effective tool in a wide range of scientific and engineering problems. Among all the definitions of fractional derivative that have been introduced so far, the Atangana beta derivative is more reliable.

## Outline of the IBSEF method

The BSEFM has been extended to derive the IBSEF method^21^, which will be detailed in this section.

**Step I.** Let’s consider the following equation for the fractional differential equation:

8 $${\mathcal{R}}\left( {D_{t}^{\gamma } u,{ }u_{x} ,u_{t} ,u_{xt} , \ldots } \right) = 0,$$

where $u = u\left( {x,t} \right)$ is a function which is not defined, and $\gamma \in \left( {0, 1} \right]$ is the order of beta-derivative.

Using the following wave transformation

9 $$u\left( {x,t} \right) = U\left( \eta \right),\;\;\eta = \frac{m}{\gamma }\left( {x + \frac{1}{{{{\Gamma \gamma }}}}} \right)^{\gamma } + \frac{k}{\gamma }\left( {t + \frac{1}{{{{\Gamma \gamma }}}}} \right)^{\gamma } ,$$

where $m$ and $k$ are the wave number and velocity respectively and $0<\gamma \le 1$ indicates fractional-order. It can be converted Eq. (8) into nonlinear ordinary differential equation (NLODE) based on the above transformation (9) as:

10 $${\mathcal{S}}\left( {U, U^{\prime},U^{\prime\prime},U^{\prime\prime\prime}, \ldots } \right) = 0.$$

**Step II.** According to the IBSEF method, the trial solution in Eq. (10) can be expressed as

11 $$U\left( \eta \right) = \frac{{\mathop \sum \nolimits_{i = 0}^{l} p_{i} {\mathcal{H}}^{i} \left( \eta \right)}}{{\mathop \sum \nolimits_{j = 0}^{m} q_{j} {\mathcal{H}}^{j} \left( \eta \right)}} = \frac{{p_{0} + p_{1} {\mathcal{H}}\left( \eta \right) + p_{2} {\mathcal{H}}^{2} \left( \eta \right) + \cdots + p_{l} {\mathcal{H}}^{l} \left( \eta \right)}}{{q_{0} + q_{1} {\mathcal{H}}\left( \eta \right) + q_{2} {\mathcal{H}}^{2} \left( \eta \right) + \cdots + q_{m} {\mathcal{H}}^{m} \left( \eta \right)}}.$$

In accordance with the Bernoulli theory, we can assume the general form of Bernoulli differential equation for ${\mathcal{H}^{\prime}}$ as underneath:

12 $${\mathcal{H}^{\prime}} = \alpha {\mathcal{H}} + \beta {\mathcal{H}}^{{\mathcal{N}}} ,\;\;\alpha , \beta \ne 0,{\mathcal{N}} \in {\mathbb{R}} - \left\{ {0, 1, 2} \right\},$$

where $\mathcal{H}=\mathcal{H}\left(\eta \right)$ is the solution of Eq. (12)

Substituting the solution (11) into the Eq. (10) and making use of the Eq. (12), it gives the following polynomial ${\Omega }\left( {\mathcal{H}} \right)$ of ${\mathcal{H}}$:

13 $${\Omega }\left( {\mathcal{H}} \right) = n_{s} {\mathcal{H}}^{s} + \cdots n_{1} {\mathcal{H}}^{1} + n_{0} = 0.$$

To determine the values of $l, m$ and $\mathcal{N}$, where $l$ and $m$ both are unknowns, we use homogeneous balancing technique of the largest order linear term with the highest order nonlinear term.

**Step III.** By setting the coefficients of $\Omega \left(\mathcal{H}\right)$ to zero, we can obtain a system of equations:

14 $$n_{l} = 0, l = 0, \ldots , s.$$

We must solve the system in order to find the values of $p_{0} ,p_{1} ,p_{2} , \ldots ,p_{d}$ and $q_{0} ,q_{1} ,q_{2} , \ldots ,q_{c}$.

**Step IV.** The following couple of outcomes that we obtain from solving nonlinear BDE reliant on the values of the parameters $\alpha$ and $\beta$:

15 $${\mathcal{H}}\left( \eta \right) = \left[ { - \frac{\beta }{\alpha } + \frac{\tau }{{e^{{\left[ {\alpha \left( {{\mathcal{N}} - 1} \right)\eta } \right]}} }}} \right]^{{\frac{1}{{\left( {1 - {\mathcal{N}}} \right)}}}} ,\;{\text{for}}\;\alpha \ne \beta ,$$

16 $${\mathcal{H}}\left( \eta \right) = \left[ {\frac{{\left( {\tau - 1} \right) + \left( {\tau + 1} \right){\text{tanh}}\left( {\frac{{\alpha \left( {1 - {\mathcal{N}}} \right)\eta }}{2}} \right)}}{{1 - \tanh \left( {\frac{{\alpha \left( {1 - {\mathcal{N}}} \right)\eta }}{2}} \right)}}} \right]^{{\frac{1}{{\left( {1 - {\mathcal{N}}} \right)}}}} ,\;\;{\text{for}}\;\,\alpha = \beta , \tau \in {\mathbb{R}}.$$

**Advantages:** The approach has advantages over other methods, such as the exp-function method, tanh-function method, basic $\left( {G^{\prime} /G} \right)$-expansion method, etc. The suggested approach offers further exact traveling wave solutions with extra free parameters. These precise solutions are essential for revealing the underlying principles of physical phenomena. When applicable, the IBSEF method can yield closed-form solutions to space–time fractional equations, which provide a clear mathematical expression for the behavior of the system. On the other hand, when dealing with reduced differential equations of third order or below, using symbolic computation software like Maple substantially enhances the possibility of obtaining useful solutions to the related algebraic equations. However, it gets more difficult to guarantee the existence of solutions for the resultant algebraic equations in general as the order of the equations rises. The capacity of the recommended approach to accommodate a greater number of arbitrary constants than other existing methods makes them particularly useful in such circumstances. This strategy can solve problems where other approaches could fail thanks to this quality.

**Limitations:** The method has a limitation that should be taken into account despite its many benefits. It occasionally creates solutions that are disguised versions of well-known solutions that may be found using other techniques. Additionally, the approach cannot guarantee that there are solutions to the resultant algebraic equations when the balance number of the reduced ordinary differential equations is greater than four.

## Analysis of closed form solutions

By using the definition of beta derivative, we determined some advanced, broad-ranging and further inclusive closed form stable soliton solutions to the space–time fractional NLKG equation and mRLW equation with the help of IBSEF method.

### The space–time fractional nonlinear Klein–Gordon equation

In 1926, the famous physicists Klein^40^ and Gordon^41^ first introduced the space–time fractional NLKG equation. Now consider this equation as follows^42^:

17 $$D_{t}^{2\gamma } V - \nu D_{x}^{2\gamma } V + aV - bV^{3} = 0; \quad 0\,\,\left\langle {\gamma \le 1, t} \right\rangle\,\, 0,$$

where the constants $a$, $\nu$ and $b$ are non-zero coefficients depending upon physical conditions of the system. These constants will be evaluated afterwards through the computational package Maple which will represent numerous phenomena of the solutions. Also, $\gamma$ denotes a derivative whose order is fractional.

Applying the wave transformation

18 $$\eta = \frac{m}{\gamma }\left( {x + \frac{1}{{{{\Gamma \gamma }}}}} \right)^{\gamma } + \frac{k}{\gamma }\left( {t + \frac{1}{{{{\Gamma \gamma }}}}} \right)^{\gamma } ,\;\;V\left( {x,t} \right) = V\left( \eta \right),$$

where $m$ be the number of waves, $k$ be the wave speed, and $\eta$ represents wave transformation.

Substituting Eq. (18) into Eq. (17), then Eq. (17) can be transformed to an integer order NLODE as follows:

19 $$\left( {k^{2} - \nu m^{2} } \right)V^{\prime\prime} + aV - bV^{3} = 0.$$

For the maximum order linear term $V^{\left( \omega \right)}$, the exponent of $V$ is $\omega {\mathcal{N}} + d - c - 1$ and for the maximum degree nonlinear term $V^{n}$ whose exponent is $n\left( {d - c} \right) + 1$. Since, soliton solutions are being sought, and thus the exponent of the linear term is identical to that of the nonlinear term. After balancing^43, 44^ these two terms we obtain $\omega {\mathcal{N}} + d - c - 1$ = $n\left( {d - c} \right) + 1$*.* From Eq. (19), we observe that $V^{3} \left( {n = 3} \right)$ is the nonlinear term of highest degree as well as the linear term of maximum order is $V^{\prime\prime}\left( {\omega = 2} \right)$. Now, using these values of $\omega$ and $n$ we get the relationship shown below:

$$d + 1 = c + {\mathcal{N}}.$$

Here ${\mathcal{N}}$ and $c$ are random parameters, selecting $c = 1$ and ${\mathcal{N}} = 3$, which leads to $d = 3$. Therefore, the general solution is provided by the trial solution to Eq. (19) in the following way:

20 $$V\left( \eta \right) = \frac{{p_{0} + p_{1} {\mathcal{H}}\left( \eta \right) + p_{2} {\mathcal{H}}^{2} \left( \eta \right) + p_{3} {\mathcal{H}}^{3} \left( \eta \right)}}{{q_{0} + q_{1} {\mathcal{H}}\left( \eta \right)}},$$

where ${\mathcal{H}}$ will be calculated from the IBSEF method.

Now, to determine $V^{\prime}\left( \eta \right)$ and $V^{\prime\prime}\left( \eta \right)$, we can write as follows:

21 $$V\left( \eta \right) = \frac{{p_{0} + p_{1} {\mathcal{H}}\left( \eta \right) + p_{2} {\mathcal{H}}^{2} \left( \eta \right) + p_{3} {\mathcal{H}}^{3} \left( \eta \right)}}{{q_{0} + q_{1} {\mathcal{H}}\left( \eta \right)}} = \frac{\psi \left( \eta \right)}{{\varphi \left( \eta \right)}} ,$$

22 $$V^{\prime}\left( \eta \right) = \frac{{\psi^{\prime}\left( \eta \right)\varphi \left( \eta \right) - \psi \left( \eta \right)\varphi ^{\prime}\left( \eta \right)}}{{\varphi^{2} \left( \eta \right)}} ,$$

and

23 $$V^{\prime\prime}\left( \eta \right) = \frac{{\psi^{\prime\prime}\left( \eta \right)\varphi \left( \eta \right) - \psi \left( \eta \right)\varphi^{\prime}\left( \eta \right)}}{{\varphi^{2} \left( \eta \right)}} - \frac{{\left[ {\psi \left( \eta \right)\varphi ^{\prime}\left( \eta \right)} \right]^{\prime}\varphi^{2} \left( \eta \right) - 2\psi \left( \eta \right)\left[ {\varphi ^{\prime}\left( \eta \right)} \right]^{2} \varphi \left( \eta \right)}}{{\varphi^{4} \left( \eta \right)}} ,$$

where ${\mathcal{H}^{\prime}} = \alpha {\mathcal{H}} + \beta {\mathcal{H}}^{{\mathcal{N}}} ,$ $\alpha \ne 0, \beta \ne 0.$ Eqs. (21 and 23) are used in Eq. (19) to produce a system of algebraic equations based on the coefficients of ${\mathcal{H}}$ polynomial. By solving the system of algebraic equations with the use of Maple 13, the constant values may be determined.

**Set 1.**

24 $$k = \frac{{\sqrt {4\alpha^{2} m^{2} \nu + 2a} }}{2\alpha }, \;\; p_{0} = \frac{{aq_{0} }}{{\sqrt {ba} }}, p_{1} = 0, \;\; p_{2} = \frac{{2q_{0} \beta \sqrt {ba} }}{b\alpha }, \;\;p_{3} = 0, \;\; q_{1} = 0.$$

**Case 1a:** When $$\alpha \ne \beta$$

By using the parameter values accumulated in (24) alongside (15) into the solution (20), we achieve the exponential function solution (EFS) to the space–time fractional NLKG equation written as:

25 $$V_{{1_{1} }} \left( {x,t} \right) = \frac{1}{{q_{0} }}\left\{ { \frac{{aq_{0} }}{{\sqrt {ba} }} + \frac{{2q_{0} \beta \sqrt {ba} }}{{b\alpha \left( { - \frac{\beta }{\alpha } + \frac{\tau }{{e^{{2\alpha \left( {\frac{m}{\gamma }\left( {x + \frac{1}{{{{\Gamma \gamma }}}}} \right)^{\gamma } + \frac{k}{\gamma }\left( {t + \frac{1}{{{{\Gamma \gamma }}}}} \right)^{\gamma } } \right)}} }}} \right)}}} \right\},$$

where the constants $a$, $b$, $m$, $k$ are non-zero.

**Case 1b:** When $$\alpha =\beta$$

Using the values of the coefficients accumulated in (24) alongside (16) into the solution (20), we achieve the hyperbolic trigonometric function solution (HTFS) to the space–time fractional NLKG equation given below:

26 $$V_{{1_{2} }} \left( {x,t} \right) = \frac{1}{{q_{0} }} \left\{ {\frac{{aq_{0} }}{{\sqrt {ba} }} + \frac{{2q_{0} \sqrt {ba} \left( {1 + \tanh \left( {\beta \left( {\frac{m}{\gamma }\left( {x + \frac{1}{{{{\Gamma \gamma }}}}} \right)^{\gamma } + \frac{k}{\gamma }\left( {t + \frac{1}{{{{\Gamma \gamma }}}}} \right)^{\gamma } } \right)} \right)} \right)}}{{b\left( {\tau - 1 - \left( {\tau + 1} \right)\tanh \left( {\beta \left( {\frac{m}{\gamma }\left( {x + \frac{1}{{{{\Gamma \gamma }}}}} \right)^{\gamma } + \frac{k}{\gamma }\left( {t + \frac{1}{{{{\Gamma \gamma }}}}} \right)^{\gamma } } \right)} \right)} \right)}}} \right\},$$

where the constants $a$, $b$, $m$, $k$ are non-zero.

**Set 2.**

27 $$k = \frac{{\sqrt {4\alpha^{2} m^{2} \nu + 2a} }}{2\alpha }, \;\;p_{0} = \frac{{\alpha p_{2} }}{2\beta },\;\; p_{1} = \frac{{aq_{1} }}{{\sqrt {ba} }},\;\; p_{3} = \frac{{2q_{1} \beta \sqrt {ba} }}{b\alpha }, \;\; q_{0} = \frac{{b\alpha p_{2} }}{{2\beta \sqrt {ba} }}.$$

**Case 2a:** When $$\alpha \ne \beta$$.

If we take the Eq. (27) coefficients along with Eq. (15) in Eq. (20), we find the EFS to the NLKG equation given as following:

28 $${V}_{{1}_{3}}\left(x,t\right)=\frac{\begin{array}{c}\frac{\alpha {p}_{2}}{2\beta } + \frac{{q}_{1}a}{\sqrt{ba}\sqrt{\left(- \frac{\beta }{\alpha } + \frac{\tau }{{e}^{2\alpha \left({\frac{m}{\gamma }\left(x + \frac{1}{\mathrm{\Gamma \gamma }}\right)}^{\gamma }+ {\frac{k}{\gamma }\left(t + \frac{1}{\mathrm{\Gamma \gamma }}\right)}^{\gamma }\right)}}\right)}} +\\ \frac{{p}_{2}}{\left(- \frac{\beta }{\alpha } + \frac{\tau }{{e}^{2\alpha \left({\frac{m}{\gamma }\left(x + \frac{1}{\mathrm{\Gamma \gamma }}\right)}^{\gamma }+ {\frac{k}{\gamma }\left(t + \frac{1}{\mathrm{\Gamma \gamma }}\right)}^{\gamma }\right)}}\right)} + \frac{2\sqrt{ba}\beta {q}_{1}}{b\alpha {\left(- \frac{\beta }{\alpha } + \frac{\tau }{{e}^{2\alpha \left({\frac{m}{\gamma }\left(x + \frac{1}{\mathrm{\Gamma \gamma }}\right)}^{\gamma }+ {\frac{k}{\gamma }\left(t + \frac{1}{\mathrm{\Gamma \gamma }}\right)}^{\gamma }\right)}}\right)}^\frac{3}{2}}\end{array}}{\frac{\alpha b{p}_{2}}{2\sqrt{ba}\beta } + \frac{{q}_{1}}{\sqrt{\left(- \frac{\beta }{\alpha } + \frac{\tau }{{e}^{2\alpha \left({\frac{m}{\gamma }\left(x + \frac{1}{\mathrm{\Gamma \gamma }}\right)}^{\gamma }+ {\frac{k}{\gamma }\left(t + \frac{1}{\mathrm{\Gamma \gamma }}\right)}^{\gamma }\right)}}\right)}}}.$$

**Case 2b**: When $$\alpha =\beta$$

If we take the Eq. (27) coefficients along with Eq. (16) in Eq. (20), we find the HTFS shown as:

29 $${V}_{{1}_{4}}\left(x,t\right)=\frac{\begin{array}{c}\frac{{p}_{2}}{2} + \frac{{q}_{1}a}{\sqrt{ba}\sqrt{\left(\frac{\tau -1-\left(\tau + 1\right)\mathrm{tanh}\left(\beta \left({\frac{m}{\gamma }\left(x + \frac{1}{\mathrm{\Gamma \gamma }}\right)}^{\gamma }+ {\frac{k}{\gamma }\left(t + \frac{1}{\mathrm{\Gamma \gamma }}\right)}^{\gamma }\right)\right)}{1 +\mathrm{ tanh}\left(\beta \left({\frac{m}{\gamma }\left(x + \frac{1}{\mathrm{\Gamma \gamma }}\right)}^{\gamma }+ {\frac{k}{\gamma }\left(t + \frac{1}{\mathrm{\Gamma \gamma }}\right)}^{\gamma }\right)\right)}\right)}} +\\ \frac{{p}_{2}\left(1 + \mathrm{tanh}\left(\beta \left({\frac{m}{\gamma }\left(x + \frac{1}{\mathrm{\Gamma \gamma }}\right)}^{\gamma }+ {\frac{k}{\gamma }\left(t + \frac{1}{\mathrm{\Gamma \gamma }}\right)}^{\gamma }\right)\right)\right)}{\left(\tau -1-\left(\tau + 1\right)\mathrm{tanh}\left(\beta \left({\frac{m}{\gamma }\left(x + \frac{1}{\mathrm{\Gamma \gamma }}\right)}^{\gamma }+ {\frac{k}{\gamma }\left(t + \frac{1}{\mathrm{\Gamma \gamma }}\right)}^{\gamma }\right)\right)\right)} + \frac{2\sqrt{ba}{q}_{1}}{b{\left(\frac{\tau -1-\left(\tau + 1\right)\mathrm{tanh}\left(\beta \left({\frac{m}{\gamma }\left(x + \frac{1}{\mathrm{\Gamma \gamma }}\right)}^{\gamma }+ {\frac{k}{\gamma }\left(t + \frac{1}{\mathrm{\Gamma \gamma }}\right)}^{\gamma }\right)\right)}{1 + \mathrm{tanh}\left(\beta \left({\frac{m}{\gamma }\left(x + \frac{1}{\mathrm{\Gamma \gamma }}\right)}^{\gamma }+ {\frac{k}{\gamma }\left(t + \frac{1}{\mathrm{\Gamma \gamma }}\right)}^{\gamma }\right)\right)}\right)}^\frac{3}{2}}\end{array}}{\frac{b{p}_{2}}{2\sqrt{ba}} + \frac{{q}_{1}}{\sqrt{\left(\frac{\tau -1-\left(\tau +1\right)\mathrm{tanh}\left(\beta \left({\frac{m}{\gamma }\left(x + \frac{1}{\mathrm{\Gamma \gamma }}\right)}^{\gamma }+ {\frac{k}{\gamma }\left(t + \frac{1}{\mathrm{\Gamma \gamma }}\right)}^{\gamma }\right)\right)}{1 + \mathrm{tanh}\left(\beta \left({\frac{m}{\gamma }\left(x + \frac{1}{\mathrm{\Gamma \gamma }}\right)}^{\gamma }+ {\frac{k}{\gamma }\left(t + \frac{1}{\mathrm{\Gamma \gamma }}\right)}^{\gamma }\right)\right)}\right)}}}.$$

**Set 3.**

30 $$p_{0} = \frac{{q_{0} \sqrt {ba} }}{b}, \;\; p_{1} = \frac{{q_{1} \sqrt {ba} }}{b},\;\; p_{2} = 0 = p_{3} .$$

**Case 3a:** When $$\alpha \ne \beta$$.

Putting Eq. (30) along with Eq. (15) in Eq. (20), we acquire the EFS to the NLKG equation written as:

31 $$V_{{1_{5} }} \left( {x,t} \right) = \frac{{\frac{{\sqrt {ba} q_{0} }}{b} + \frac{{q_{1} \sqrt {ba} }}{{b\left( {\sqrt { - \frac{\beta }{\alpha } + \frac{\tau }{{e^{{2\alpha \left( {\frac{m}{\gamma }\left( {x + \frac{1}{{{{\Gamma \gamma }}}}} \right)^{\gamma } + \frac{k}{\gamma }\left( {t + \frac{1}{{{{\Gamma \gamma }}}}} \right)^{\gamma } } \right)}} }}} } \right)}}}}{{q_{0} + \frac{{q_{1} }}{{\left( {\sqrt { - \frac{\beta }{\alpha } + \frac{\tau }{{e^{{2\alpha \left( {\frac{m}{\gamma }\left( {x + \frac{1}{{{{\Gamma \gamma }}}}} \right)^{\gamma } + \frac{k}{\gamma }\left( {t + \frac{1}{{{{\Gamma \gamma }}}}} \right)^{\gamma } } \right)}} }}} } \right)}}}}.$$

**Case 3b:** When $$\alpha =\beta$$

Putting Eq. (30) along with Eq. (16) in Eq. (20), we acquire the HTFS to the NLKG equation given below:

32 $${V}_{{1}_{6}}\left(x,t\right)=\frac{\frac{\sqrt{ba}{q}_{0}}{b} + \frac{{q}_{1}\sqrt{ba}}{b\left(\sqrt{\frac{\tau -1-\left(\tau + 1\right)\mathrm{tanh}\left(\beta \left({\frac{m}{\gamma }\left(x + \frac{1}{\mathrm{\Gamma \gamma }}\right)}^{\gamma }+ {\frac{k}{\gamma }\left(t + \frac{1}{\mathrm{\Gamma \gamma }}\right)}^{\gamma }\right)\right)}{1 + \mathrm{tanh}\left(\beta \left({\frac{m}{\gamma }\left(x + \frac{1}{\mathrm{\Gamma \gamma }}\right)}^{\gamma }+ {\frac{k}{\gamma }\left(t + \frac{1}{\mathrm{\Gamma \gamma }}\right)}^{\gamma }\right)\right)}}\right)}}{{q}_{0}+\frac{{q}_{1}}{\left(\sqrt{\frac{\tau -1-\left(\tau + 1\right)\mathrm{tanh}\left(\beta \left({\frac{m}{\gamma }\left(x + \frac{1}{\mathrm{\Gamma \gamma }}\right)}^{\gamma }+ {\frac{k}{\gamma }\left(t + \frac{1}{\mathrm{\Gamma \gamma }}\right)}^{\gamma }\right)\right)}{1 + \mathrm{tanh}\left(\beta \left({\frac{m}{\gamma }\left(x + \frac{1}{\mathrm{\Gamma \gamma }}\right)}^{\gamma }+ {\frac{k}{\gamma }\left(t + \frac{1}{\mathrm{\Gamma \gamma }}\right)}^{\gamma }\right)\right)}}\right)}}.$$

All the general solutions to the space–time fractional NLKG equation presented here are valid and effective, and interestingly, all of these solutions have not been previously investigated.

### The space–time fractional modified regularized long-wave equation

The space–time fractional mRLW equation was initially deduced by Benjamin^45^ in 1972 that explains generally the one-way propagation of long waves in certain nonlinear dispersive systems. This equation is regarded as a substitute for the Korteweg–de Vries equation, which is designed to describe numerous physical phenomena like plasma waves and shallow waters in coastal oceans. The space–time fractional mRLW equation is taken into consideration as^32^:

33 $$D_{t}^{\gamma } V + \zeta D_{x}^{\gamma } V + \varepsilon V^{2} D_{x}^{\gamma } V - \mu D_{xxt}^{3\gamma } V = 0; 0\,\, \langle {\gamma \le 1,t} \rangle \,\,0,$$

where $\gamma$ is a fractional derivative, and $\zeta ,$ $\varepsilon ,$ and $\mu$ are arbitrary constants depend on the system's physical properties. Following that, these constants will be assessed using the computational tool Maple, which will represent a variety of phenomena of the solutions. Using the complex fractional wave variable

34 $$\eta = \frac{m}{\gamma }\left( {x + \frac{1}{{{{\Gamma \gamma }}}}} \right)^{\gamma } + \frac{k}{\gamma }\left( {t + \frac{1}{{{{\Gamma \gamma }}}}} \right)^{\gamma } ,\;V\left( {x,t} \right) = V\left( \eta \right),$$

where $m$ be the number of waves, $k$ be the wave speed, and $\eta$ represents wave transformation. Then the space–time fractional mRLW Eq. (24) is transformed into an integer order NLODE as follows:

35 $$kV^{\prime} + \zeta mV^{\prime} + \varepsilon V^{2} mV^{\prime} - \mu km^{2} V^{\prime\prime\prime} = 0.$$

After integrating Eq. (25) with respect to $\eta$ once then putting integrating constant $= 0$, we get the equation of following form:

36 $$kV + \zeta mV + \varepsilon mV^{3} - \mu km^{2} V^{\prime\prime} = 0.$$

Rewrite Eq. (26) as

37 $$\left( {k + \zeta m} \right)V + \varepsilon mV^{3} - \mu km^{2} V^{\prime\prime} = 0.$$

As the relationship among $d$, $c$, and ${\mathcal{N}}$ is covered in detail in section “The space–time fractional nonlinear Klein–Gordon equation”, so it is not extensively outlined here. From Eq. (27), we observe that $V^{\prime\prime}$ is the largest order derivative term and $V^{3}$ is the nonlinear term of height order. Consequently, a relationship for $d$, $c$, and ${\mathcal{N}}$ is obtained by balancing the above-mentioned two maximum order terms in Eq. (27), which can be expressed in this way:

$$c + {\mathcal{N}} = d + 1.$$

Taking ${\mathcal{N}} = 3$, $c = 1$ gives $d = 3$ and putting these values into Eq. (11) which provides the trial solution of Eq. (27) in the ensuing structure:

38 $$V\left( \eta \right) = \frac{{p_{0} + p_{1} {\mathcal{H}}\left( \eta \right) + p_{2} {\mathcal{H}}^{2} \left( \eta \right) + p_{3} {\mathcal{H}}^{3} \left( \eta \right)}}{{q_{0} + q_{1} {\mathcal{H}}\left( \eta \right)}},$$

where $\mathcal{H}$ is the root of Eq. (12)

Setting the solution (38), alongside with (12) into (37), yields a polynomial in $\mathcal{H}$. A set of equations for the parameters can be obtained, and by solving the system of algebraic equations using Maple 13, the constant values can be determined.

**Family 1.**

39 $$k = \frac{{\varepsilon p_{2} \left( {\varepsilon \alpha^{2} p_{2}^{2} + 4\zeta \beta^{2} q_{0}^{2} } \right)}}{{4\beta^{2} q_{0}^{2} \sqrt { - 2\mu \left( {\varepsilon \alpha^{2} p_{2}^{2} + 4\zeta \beta^{2} q_{0}^{2} } \right)\varepsilon } }}, \;\; m = \frac{{\sqrt { - 2\mu \left( {\varepsilon \alpha^{2} p_{2}^{2} + 4\zeta \beta^{2} q_{0}^{2} } \right)\varepsilon } p_{2} }}{{2\mu \left( {\varepsilon \alpha^{2} p_{2}^{2} + 4\zeta \beta^{2} q_{0}^{2} } \right)}}, \;\; p_{0} = \frac{{\alpha p_{2} }}{2\beta }, \;\; p_{1} = 0, \;\; p_{3} = 0, \;\;q_{1} = 0.$$

**Category 1a:** First, we assume $\alpha \ne \beta$, because the solutions to the IBSEF approach improved Bernoulli equation depend on these two variables.

By entering the listed parameters values in (39) along with (15) into solution (38) provides the EFS written as:

40 $$V_{{2_{1} }} \left( {x,t} \right) = \frac{1}{{q_{0} }} \left\{ {\frac{{\alpha p_{2} }}{2\beta } + \frac{{p_{2} }}{{\left( { - \frac{\beta }{\alpha } + \frac{\tau }{{e^{{2\alpha \left( {\frac{m}{\gamma }\left( {x + \frac{1}{{{{\Gamma \gamma }}}}} \right)^{\gamma } + \frac{k}{\gamma }\left( {t + \frac{1}{{{{\Gamma \gamma }}}}} \right)^{\gamma } } \right)}} }}} \right)}}} \right\},$$

After simplifying, from (40), we can write

41 $$V_{{2_{2} }} \left( {x,t} \right) = \frac{1}{{q_{0} }} \left\{ {\frac{{\alpha p_{2} }}{2\beta } + \frac{{p_{2} }}{{\left( { - \frac{\beta }{\alpha } + \frac{\tau }{{cos\left( {2\alpha \left( {\frac{m}{\gamma }\left( {x + \frac{1}{{{{\Gamma \gamma }}}}} \right)^{\gamma } + \frac{k}{\gamma }\left( {t + \frac{1}{{{{\Gamma \gamma }}}}} \right)^{\gamma } } \right)} \right) + isin\left( {2\alpha \left( {\frac{m}{\gamma }\left( {x + \frac{1}{{{{\Gamma \gamma }}}}} \right)^{\gamma } + \frac{k}{\gamma }\left( {t + \frac{1}{{{{\Gamma \gamma }}}}} \right)^{\gamma } } \right)} \right)}}} \right)}}} \right\}.$$

**Category 1b:** Consider $$\alpha =\beta$$

By entering the listed parameters values in (39) along with (16) into solution (38) provides the HTFS in this way:

42 $$V_{{2_{3} }} \left( {x,t} \right) = \frac{1}{{q_{0} }}\left\{ {\frac{{p_{2} }}{2} + \frac{{\left( {1 + tanh\left( {\beta \left( {\frac{m}{\gamma }\left( {x + \frac{1}{{{{\Gamma \gamma }}}}} \right)^{\gamma } + \frac{k}{\gamma }\left( {t + \frac{1}{{{{\Gamma \gamma }}}}} \right)^{\gamma } } \right)} \right)} \right)p_{2} }}{{\left( {\tau - 1 - \left( {\tau + 1} \right)\tanh \left( {\beta \left( {\frac{m}{\gamma }\left( {x + \frac{1}{{{{\Gamma \gamma }}}}} \right)^{\gamma } + \frac{k}{\gamma }\left( {t + \frac{1}{{{{\Gamma \gamma }}}}} \right)^{\gamma } } \right)} \right)} \right)}}} \right\} .$$

Apply trigonometric formula in Eq. (32),

43 $$V_{{2_{4} }} \left( {x,t} \right) = \frac{1}{{q_{0} }}\left\{ {\frac{{p_{2} }}{2}\frac{{\left( {1 + \left( {\frac{{sinh\left( {\beta \left( {\frac{m}{\gamma }\left( {x + \frac{1}{{{{\Gamma \gamma }}}}} \right)^{\gamma } + \frac{k}{\gamma }\left( {t + \frac{1}{{{{\Gamma \gamma }}}}} \right)^{\gamma } } \right)} \right)}}{{cosh\left( {\beta \left( {\frac{m}{\gamma }\left( {x + \frac{1}{{{{\Gamma \gamma }}}}} \right)^{\gamma } + \frac{k}{\gamma }\left( {t + \frac{1}{{{{\Gamma \gamma }}}}} \right)^{\gamma } } \right)} \right)}}} \right)} \right)p_{2} }}{{\left( {\tau - 1 - \left( {\tau + 1} \right)\left( {\frac{{sinh\left( {\beta \left( {\frac{m}{\gamma }\left( {x + \frac{1}{{{{\Gamma \gamma }}}}} \right)^{\gamma } + \frac{k}{\gamma }\left( {t + \frac{1}{{{{\Gamma \gamma }}}}} \right)^{\gamma } } \right)} \right)}}{{cosh\left( {\beta \left( {\frac{m}{\gamma }\left( {x + \frac{1}{{{{\Gamma \gamma }}}}} \right)^{\gamma } + \frac{k}{\gamma }\left( {t + \frac{1}{{{{\Gamma \gamma }}}}} \right)^{\gamma } } \right)} \right)}}} \right)} \right)}}} \right\}.$$

**Family 2:**

44 $$k = \frac{{\varepsilon p_{3} \left( {\varepsilon \alpha^{2} p_{3}^{2} + 4\zeta \beta^{2} q_{1}^{2} } \right)}}{{4\beta^{2} q_{1}^{2} \sqrt { - 2\mu \left( {\varepsilon \alpha^{2} p_{3}^{2} + 4\zeta \beta^{2} q_{1}^{2} } \right)\varepsilon } }}, m = \frac{{\sqrt { - 2\mu \left( {\varepsilon \alpha^{2} p_{3}^{2} + 4\zeta \beta^{2} q_{1}^{2} } \right)\varepsilon } p_{3} }}{{2\mu \left( {\varepsilon \alpha^{2} p_{3}^{2} + 4\zeta \beta^{2} q_{1}^{2} } \right)}}, p_{0} = 0, p_{1} = \frac{{\alpha p_{3} }}{2\beta }, p_{2} = 0, q_{0} = 0.$$

**Phase 2a:** We take $\alpha \ne \beta$

Substituting coefficients of Eq. (33) along with Eq. (15) into (38), we find EFS as follows:

45 $${V}_{{2}_{5}}\left(x,t\right)=\frac{\begin{array}{c}\left(\frac{{\alpha p}_{3}}{2\beta \sqrt{\left(- \frac{\beta }{\alpha } + \frac{\tau }{{e}^{2\alpha \left({\frac{m}{\gamma }\left(x + \frac{1}{\mathrm{\Gamma \gamma }}\right)}^{\gamma }+ {\frac{k}{\gamma }\left(t + \frac{1}{\mathrm{\Gamma \gamma }}\right)}^{\gamma }\right)}}\right)}} + \frac{{p}_{3}}{{\left(- \frac{\beta }{\alpha } + \frac{\tau }{{e}^{2\alpha \left({\frac{m}{\gamma }\left(x + \frac{1}{\mathrm{\Gamma \gamma }}\right)}^{\gamma }+ {\frac{k}{\gamma }\left(t + \frac{1}{\mathrm{\Gamma \gamma }}\right)}^{\gamma }\right)}}\right)}^\frac{3}{2}}\right)\\ \sqrt{\left(- \frac{\beta }{\alpha } + \frac{\tau }{{e}^{2\alpha \left({\frac{m}{\gamma }\left(x + \frac{1}{\mathrm{\Gamma \gamma }}\right)}^{\gamma }+ {\frac{k}{\gamma }\left(t + \frac{1}{\mathrm{\Gamma \gamma }}\right)}^{\gamma }\right)}}\right)}\end{array}}{{q}_{1}}.$$

**Category 2b**: We take $\alpha =\beta$

Substituting coefficients of Eq. (33) along with Eq. (16) into (38), we find HTFS as follows:

46 $${V}_{{2}_{6}}\left(x,t\right)=\frac{\begin{array}{c}\left(\frac{{p}_{3}}{2\sqrt{\frac{\tau -1-\left(\tau + 1\right)\mathrm{tanh}\left(\beta \left({\frac{m}{\gamma }\left(x + \frac{1}{\mathrm{\Gamma \gamma }}\right)}^{\gamma }+ {\frac{k}{\gamma }\left(t + \frac{1}{\mathrm{\Gamma \gamma }}\right)}^{\gamma }\right)\right)}{1 + \mathrm{tanh}\left(\beta \left({\frac{m}{\gamma }\left(x + \frac{1}{\mathrm{\Gamma \gamma }}\right)}^{\gamma }+ {\frac{k}{\gamma }\left(t + \frac{1}{\mathrm{\Gamma \gamma }}\right)}^{\gamma }\right)\right)}}} + \frac{{p}_{3}}{{\left(\frac{\tau -1-\left(\tau + 1\right)\mathrm{tanh}\left(\beta \left({\frac{m}{\gamma }\left(x + \frac{1}{\mathrm{\Gamma \gamma }}\right)}^{\gamma }+ {\frac{k}{\gamma }\left(t + \frac{1}{\mathrm{\Gamma \gamma }}\right)}^{\gamma }\right)\right)}{1 + \mathrm{tanh}\left(\beta \left({\frac{m}{\gamma }\left(x + \frac{1}{\mathrm{\Gamma \gamma }}\right)}^{\gamma }+ {\frac{k}{\gamma }\left(t + \frac{1}{\mathrm{\Gamma \gamma }}\right)}^{\gamma }\right)\right)}\right)}^\frac{3}{2}}\right)\\ \sqrt{\frac{\tau -1-\left(\tau +1\right)\mathrm{tanh}\left(\beta \left({\frac{m}{\gamma }\left(x + \frac{1}{\mathrm{\Gamma \gamma }}\right)}^{\gamma }+ {\frac{k}{\gamma }\left(t + \frac{1}{\mathrm{\Gamma \gamma }}\right)}^{\gamma }\right)\right)}{1 + \mathrm{tanh}\left(\beta \left({\frac{m}{\gamma }\left(x + \frac{1}{\mathrm{\Gamma \gamma }}\right)}^{\gamma }+ {\frac{k}{\gamma }\left(t + \frac{1}{\mathrm{\Gamma \gamma }}\right)}^{\gamma }\right)\right)}}\end{array}}{{q}_{1}}.$$

**Family 3.**

47 $$k = - \frac{{\sqrt { - 2\mu \left( {\varepsilon \alpha^{2} p_{3}^{2} + 4\zeta \beta^{2} q_{1}^{2} } \right)\varepsilon } p_{3} }}{{8\mu \beta^{2} q_{1}^{2} }}, \;\; m = \frac{{\sqrt { - 2\mu \left( {\varepsilon \alpha^{2} p_{3}^{2} + 4\zeta \beta^{2} q_{1}^{2} } \right)\varepsilon } p_{3} }}{{2\mu \left( {\varepsilon \alpha^{2} p_{3}^{2} + 4\zeta \beta^{2} q_{1}^{2} } \right)}}, \;\; p_{0} = \frac{{\alpha q_{0} p_{3 } }}{{2\beta q_{1} }}, \;\; p_{1} = \frac{{\alpha p_{3} }}{2\beta }, \;\; p_{2} = \frac{{q_{0} p_{3 } }}{{q_{1} }}.$$

**Category 3a:** When $$\alpha \ne \beta$$

Using Eq. (34) along with Eq. (15) into (38), we find EFS as follows:

48 $${V}_{{2}_{7}}\left(x,t\right)=\frac{\begin{array}{c}\frac{\alpha {q}_{0} {p}_{3 }}{2\beta {q}_{1}} + \frac{{p}_{3 }\alpha }{2\beta \sqrt{\left(- \frac{\beta }{\alpha } + \frac{\tau }{{e}^{2\alpha \left({\frac{m}{\gamma }\left(x + \frac{1}{\mathrm{\Gamma \gamma }}\right)}^{\gamma }+ {\frac{k}{\gamma }\left(t + \frac{1}{\mathrm{\Gamma \gamma }}\right)}^{\gamma }\right)}}\right)}} +\\ \frac{{q}_{0} {p}_{3 }}{{q}_{1}\left(- \frac{\beta }{\alpha } + \frac{\tau }{{e}^{2\alpha \left({\frac{m}{\gamma }\left(x + \frac{1}{\mathrm{\Gamma \gamma }}\right)}^{\gamma }+ {\frac{k}{\gamma }\left(t + \frac{1}{\mathrm{\Gamma \gamma }}\right)}^{\gamma }\right)}}\right)} + \frac{{p}_{3 }}{{\left(- \frac{\beta }{\alpha } + \frac{\tau }{{e}^{2\alpha \left({\frac{m}{\gamma }\left(x + \frac{1}{\mathrm{\Gamma \gamma }}\right)}^{\gamma }+ {\frac{k}{\gamma }\left(t + \frac{1}{\mathrm{\Gamma \gamma }}\right)}^{\gamma }\right)}}\right)}^\frac{3}{2}}\end{array}}{{q}_{0} + \frac{{q}_{1}}{\sqrt{\left(- \frac{\beta }{\alpha } + \frac{\tau }{{e}^{2\alpha \left({\frac{m}{\gamma }\left(x + \frac{1}{\mathrm{\Gamma \gamma }}\right)}^{\gamma }+ {\frac{k}{\gamma }\left(t + \frac{1}{\mathrm{\Gamma \gamma }}\right)}^{\gamma }\right)}}\right)}}}.$$

**Category 3b:** When $$\alpha = \beta$$.

Using Eq. (34) along with Eq. (16) into (38), we find HTFS as follows:

49 $${V}_{{2}_{8}}\left(x,t\right)=\frac{\begin{array}{c}\frac{{q}_{0} {p}_{3 }}{{2q}_{1}} + \frac{{p}_{3}}{2\sqrt{\left(\frac{\tau -1-\left(\tau + 1\right)\mathrm{tanh}\left(\beta \left({\frac{m}{\gamma }\left(x + \frac{1}{\mathrm{\Gamma \gamma }}\right)}^{\gamma }+ {\frac{k}{\gamma }\left(t + \frac{1}{\mathrm{\Gamma \gamma }}\right)}^{\gamma }\right)\right)}{1 + \mathrm{tanh}\left(\beta \left({\frac{m}{\gamma }\left(x + \frac{1}{\mathrm{\Gamma \gamma }}\right)}^{\gamma }+ {\frac{k}{\gamma }\left(t + \frac{1}{\mathrm{\Gamma \gamma }}\right)}^{\gamma }\right)\right)}\right)}} +\\ \frac{{q}_{0} {p}_{3 }\left(1 + \mathrm{tanh}\left(\beta \left({\frac{m}{\gamma }\left(x + \frac{1}{\mathrm{\Gamma \gamma }}\right)}^{\gamma }+ {\frac{k}{\gamma }\left(t + \frac{1}{\mathrm{\Gamma \gamma }}\right)}^{\gamma }\right)\right)\right)}{{q}_{1}\left(\tau -1-\left(\tau + 1\right)\mathrm{tanh}\left(\beta \left({\frac{m}{\gamma }\left(x + \frac{1}{\mathrm{\Gamma \gamma }}\right)}^{\gamma }+ {\frac{k}{\gamma }\left(t + \frac{1}{\mathrm{\Gamma \gamma }}\right)}^{\gamma }\right)\right)\right)} + \frac{{p}_{3 }}{{\left(\frac{\tau -1-\left(\tau + 1\right)\mathrm{tanh}\left(\beta \left({\frac{m}{\gamma }\left(x + \frac{1}{\mathrm{\Gamma \gamma }}\right)}^{\gamma }+ {\frac{k}{\gamma }\left(t + \frac{1}{\mathrm{\Gamma \gamma }}\right)}^{\gamma }\right)\right)}{1 + \mathrm{tanh}\left(\beta \left({\frac{m}{\gamma }\left(x + \frac{1}{\mathrm{\Gamma \gamma }}\right)}^{\gamma }+ {\frac{k}{\gamma }\left(t + \frac{1}{\mathrm{\Gamma \gamma }}\right)}^{\gamma }\right)\right)}\right)}^\frac{3}{2}}\end{array}}{{q}_{0} + \frac{{q}_{1}}{\sqrt{\left(\frac{\tau -1-\left(\tau + 1\right)\mathrm{tanh}\left(\beta \left({\frac{m}{\gamma }\left(x + \frac{1}{\mathrm{\Gamma \gamma }}\right)}^{\gamma }+ {\frac{k}{\gamma }\left(t + \frac{1}{\mathrm{\Gamma \gamma }}\right)}^{\gamma }\right)\right)}{1 + \mathrm{tanh}\left(\beta \left({\frac{m}{\gamma }\left(x + \frac{1}{\mathrm{\Gamma \gamma }}\right)}^{\gamma }+ {\frac{k}{\gamma }\left(t + \frac{1}{\mathrm{\Gamma \gamma }}\right)}^{\gamma }\right)\right)}\right)}}}.$$

**Family 4.**

50 $$k = - \frac{{m\left( {\zeta q_{0}^{2} + \varepsilon p_{0}^{2} } \right) }}{{q_{0}^{2} }}, p_{1} = \frac{{p_{0} q_{1} }}{{q_{0} }}, p_{2} = 0 = p_{3} .$$

**Category 4a:** When $$\alpha \ne \beta$$

Using Eq. (35) along with Eq. (15) into (38), we find EFS as follows:

51 $$V_{{2_{9} }} \left( {x,t} \right) = \frac{{p_{0} + \frac{{q_{1} p_{0} }}{{q_{0} \left( {\sqrt { - \frac{\beta }{\alpha } + \frac{\tau }{{e^{{2\alpha \left( {\frac{m}{\gamma }\left( {x + \frac{1}{{{{\Gamma \gamma }}}}} \right)^{\gamma } + \frac{k}{\gamma }\left( {t + \frac{1}{{{{\Gamma \gamma }}}}} \right)^{\gamma } } \right)}} }}} } \right)}}}}{{q_{0} + \frac{{q_{1} }}{{\sqrt { - \frac{\beta }{\alpha } + \frac{\tau }{{e^{{2\alpha \left( {\frac{m}{\gamma }\left( {x + \frac{1}{{{{\Gamma \gamma }}}}} \right)^{\gamma } + \frac{k}{\gamma }\left( {t + \frac{1}{{{{\Gamma \gamma }}}}} \right)^{\gamma } } \right)}} }}} }}}}.$$

**Category 4b:** When $$\alpha =\beta$$

Using Eq. (50) along with Eq. (3.9) into (38), we find HTFS as follows:

52 $${V}_{{2}_{10}}\left(x,t\right)=\frac{{p}_{0} + \frac{{q}_{1}{p}_{0}}{{q}_{0}\left(\sqrt{\left(\frac{\tau -1-\left(\tau + 1\right)\mathrm{tanh}\left(\beta \left({\frac{m}{\gamma }\left(x + \frac{1}{\mathrm{\Gamma \gamma }}\right)}^{\gamma }+ {\frac{k}{\gamma }\left(t + \frac{1}{\mathrm{\Gamma \gamma }}\right)}^{\gamma }\right)\right)}{1 + \mathrm{tanh}\left(\beta \left({\frac{m}{\gamma }\left(x + \frac{1}{\mathrm{\Gamma \gamma }}\right)}^{\gamma }+ {\frac{k}{\gamma }\left(t + \frac{1}{\mathrm{\Gamma \gamma }}\right)}^{\gamma }\right)\right)}\right)}\right)}}{{q}_{0} + \frac{{q}_{1}}{\sqrt{\left(\frac{\tau -1-\left(\tau + 1\right)\mathrm{tanh}\left(\beta \left({\frac{m}{\gamma }\left(x + \frac{1}{\mathrm{\Gamma \gamma }}\right)}^{\gamma }+ {\frac{k}{\gamma }\left(t + \frac{1}{\mathrm{\Gamma \gamma }}\right)}^{\gamma }\right)\right)}{1 + \mathrm{tanh}\left(\beta \left({\frac{m}{\gamma }\left(x + \frac{1}{\mathrm{\Gamma \gamma }}\right)}^{\gamma }+ {\frac{k}{\gamma }\left(t + \frac{1}{\mathrm{\Gamma \gamma }}\right)}^{\gamma }\right)\right)}\right)}}}.$$

The space–time fractional mRLW equation via IBSEF method are advanced, suitable, and more flexible and all the solutions are not presented in the earlier literature.

## Results discussion and physical explanation

The attained travelling wave solutions are represented in three types of diagrams namely a three-dimensional plotline, a plot of contour, and a 2D plot for diverse values of the arbitrary constants with the help of Mathematica. The importance of graphical representations is that they can aid in improving our comprehension of solutions and analysis of complex data^46^. The diagrams depict a range of solutions namely kink wave solution, singular soliton solution, periodic soliton wave solution, bell shape, anti-bell shape, and single kink type. Now, we have shown a graphical illustration of the proposed equations.

**Figure 1:**
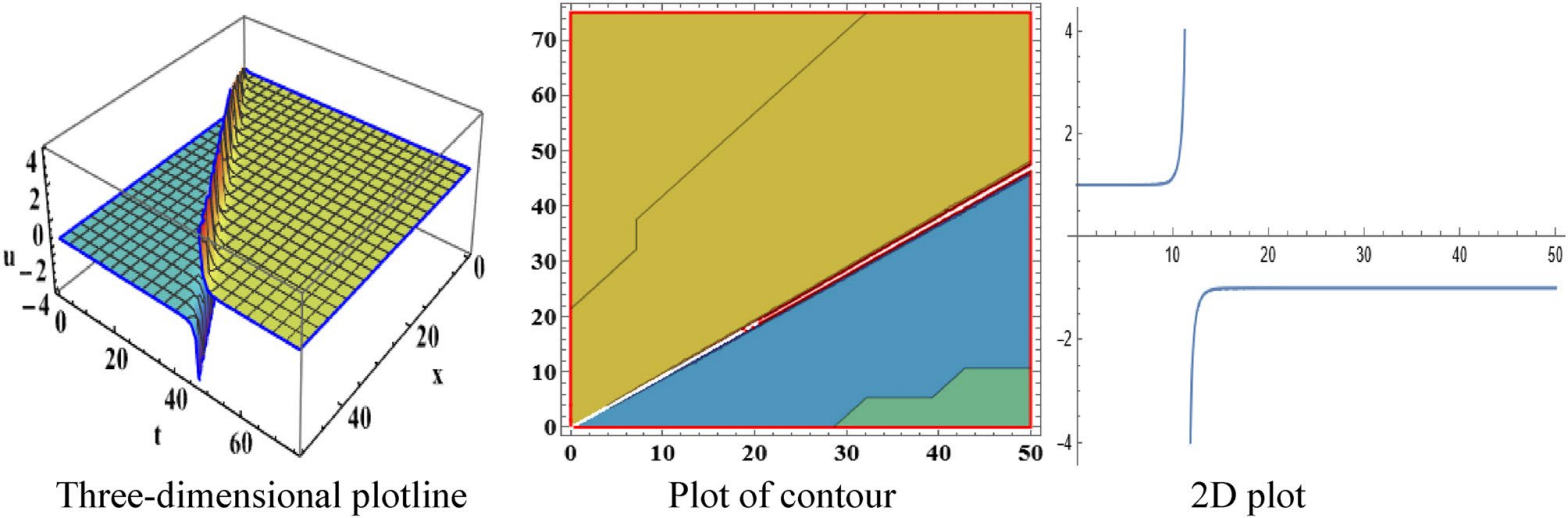
The sketch of the singular-kink shape wave solution $V_{{1_{1} }} \left( {x,t} \right),$ representing the (i) three-dimensional plotline, (ii) contour plot, and (iii) 2D plot within $0 < x < 50$, $0 < t < 50$.

**Figure 2:**
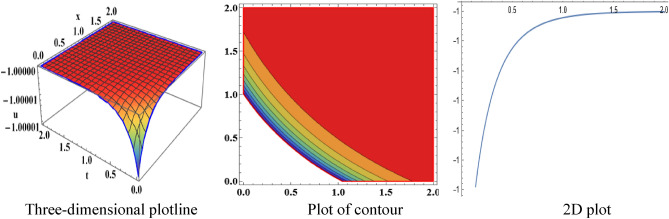
The sketch of the soliton shape wave solution $V_{{1_{2} }} \left( {x,t} \right),$ representing the (i) three-dimensional plotline, (ii) contour plot, and (iii) 2D plot within $0 < x < 2$, $0 < t < 2$.

**Figure 3:**
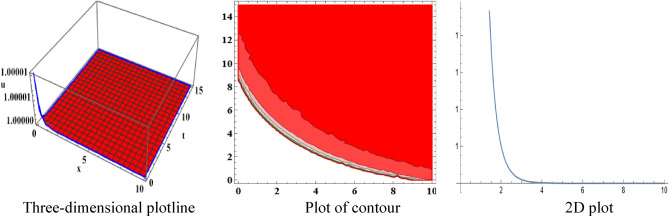
The sketch of the soliton from solution $V_{{1_{4} }} \left( {x,t} \right),$ representing the (i) three-dimensional plotline, (ii) contour plot, and (iii) 2D plot within $0 < x < 10$,$0 < t < 15$.

**Figure 4:**
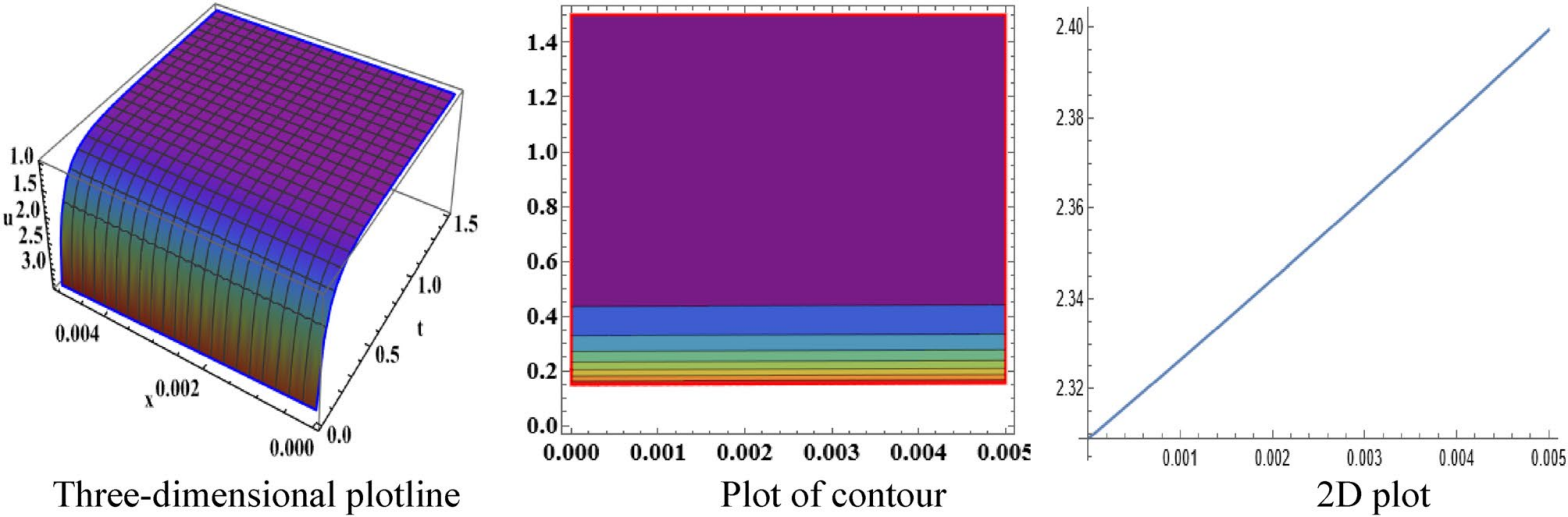
The sketch of one-sided-kink from solution $V_{{1_{3} }} \left( {x,t} \right)$, representing the (i) three-dimensional plotline, (ii) contour plot, and (iii) 2D plot within $0 < x < 0.005$, $0 < t < 1.5$.

The acquired results of the both selected equations for various parameter values are represented graphically in this section. For the values $\alpha = 3,\;\beta = 2, \;a = 1,\; b = 1$, solution $V_{{1_{1} }} \left( {x,t} \right)$ represents singular-kink shape wave featuring infinite tails in Fig. 1 within the duration $0 < x < 50$ and $0 < t < 75$. The achieved solutions which represents in $V_{{1_{2} }} \left( {x,t} \right)$ and $V_{{1_{4} }} \left( {x,t} \right)$ indicates soliton shape wave within the duration $0 < x, t < 2$, $0 < x < 10$ and $0 < t < 15$ with the same parametric values are displayed in Figs. 2 and 3. The other solution $V_{{1_{3} }} \left( {x,t} \right)$ embodies in Fig. 4 for the values of $\alpha = 3,\beta = 2, a = 1, b = 1$ within the interval $0 < x < 0.005$ and $0 < t < 1.5$ signifies one-sided-kink wave. A kink wave is a type of wave that either travels from one asymptotic position to another or rises and remains constant at infinity. It should be noted that the solution represents in $V_{{1_{5} }} \left( {x,t} \right)$ and $V_{{1_{6} }} \left( {x,t} \right)$ deliver the same types of soliton solutions but for state forwardness we discard from here.

**Figure 5:**
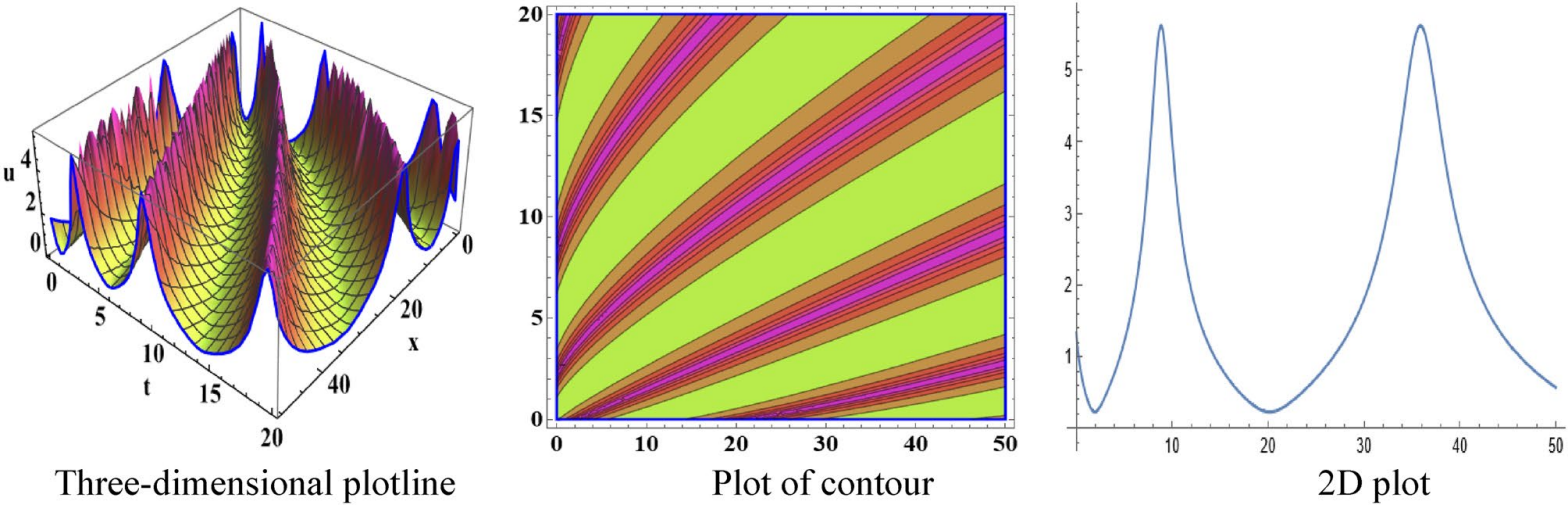
The diagram of the periodic soliton from solution $V_{{2_{2} }} \left( {x,t} \right),$ representing the (ii) three-dimensional plotline, (ii) contour plot, and (iii) 2D plot within $0 < x < 50$,$0 < t < 20$.

**Figure 6:**
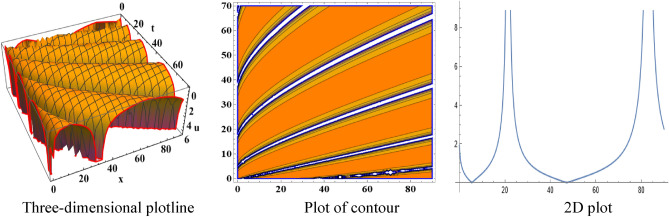
The diagram of the periodic soliton from solution $V_{{2_{4} }} \left( {x,t} \right)$, representing the (i) three-dimensional plotline, (ii) contour plot, and (iii) 2D plot within $0 < x < 90$,$0 < t < 70$.

**Figure 7:**
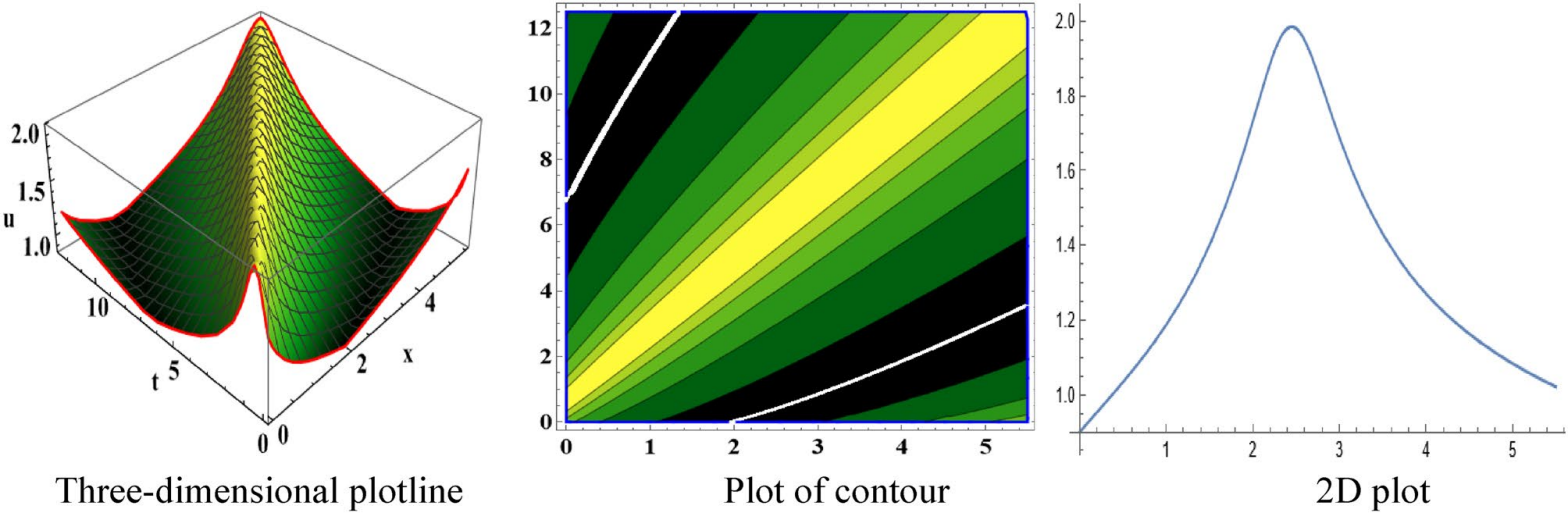
The diagram of the compacton from solution $V_{{2_{5} }} \left( {x,t} \right),$ representing the (i) three-dimensional plotline, (ii) contour plot, and (iii) 2D plot within $0 < x < 5.5$, $0 < t < 12.5$.

**Figure 8:**
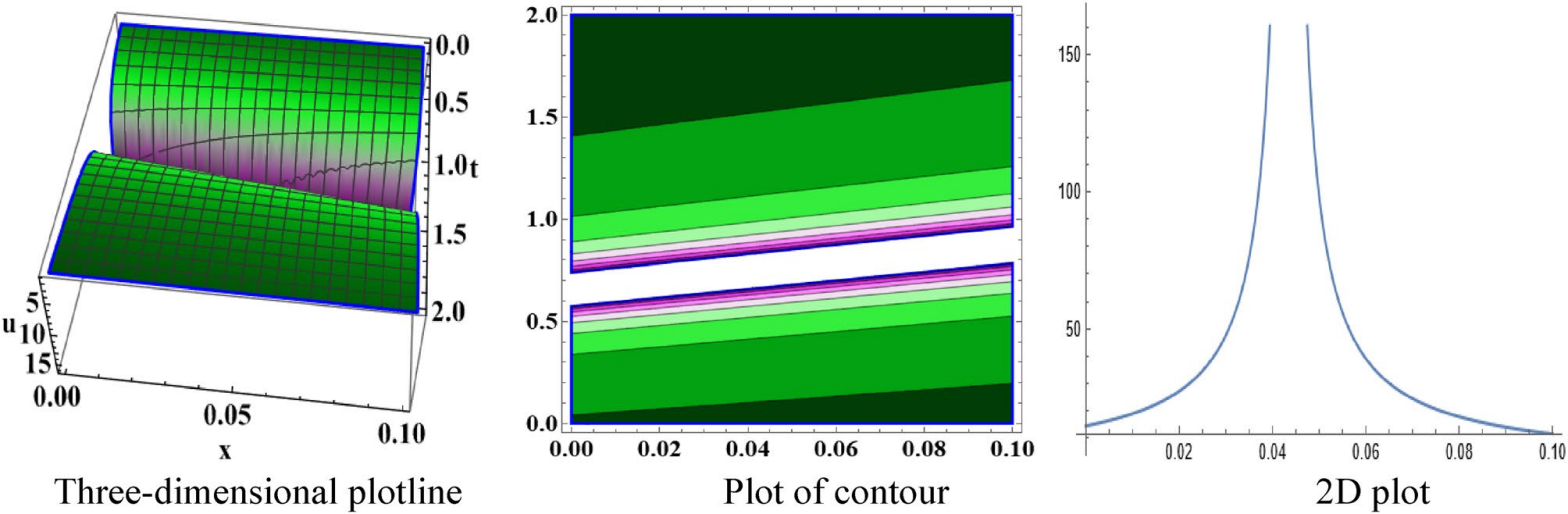
The sketch of the singular anti-bell-shaped soliton from solution $V_{{2_{6} }} \left( {x,t} \right)$, representing the (i) three-dimensional plotline, (ii) contour plot, and (iii) 2D plot within $0 < x < 0.1$, $0 < t < 2$.

**Figure 9:**
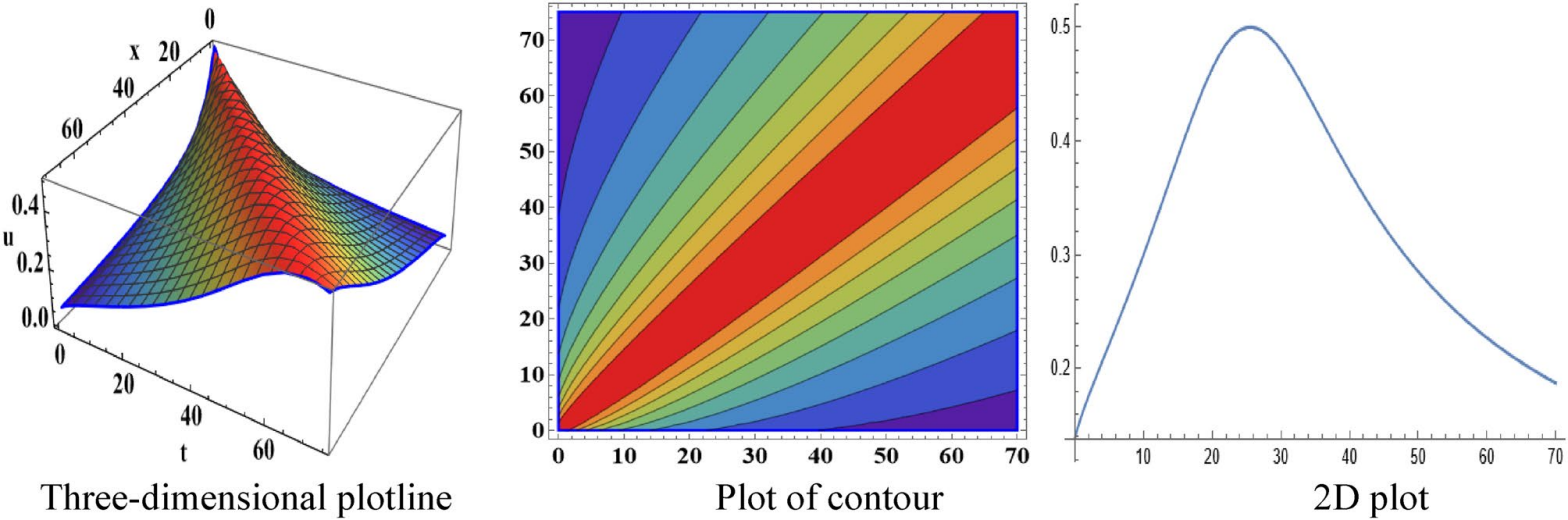
The sketch of the compacton from solution $V_{{2_{7} }} \left( {x,t} \right)$, representing (i) three-dimensional plotline, (ii) contour plot, and (iii) 2D plot within $0 < x < 70$, $0 < t < 75$.

**Figure 10:**
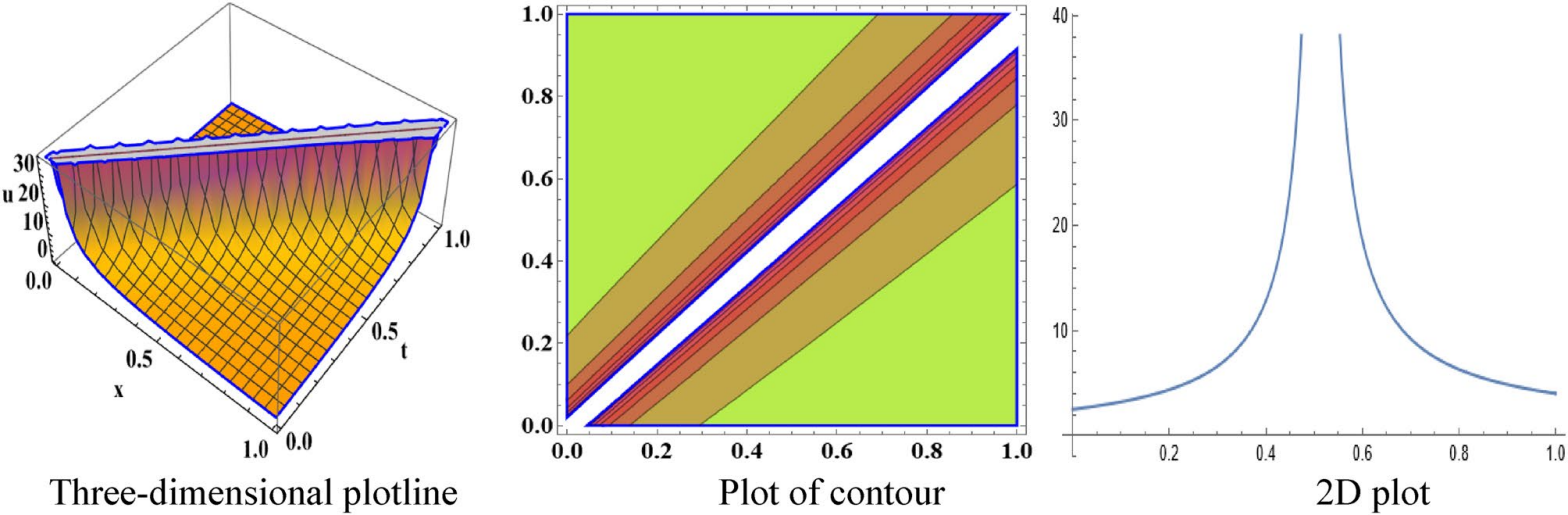
The sketch of the singular bell-shaped from solution $V_{{2_{8} }} \left( {x,t} \right)$, representing the (i) three-dimensional plotline, (ii) contour plot, and (iii) 2D plot within $0 < x < 1$, $0 < t < 1$.

In the space–time fractional mRLW equation the attained solutions $V_{{2_{2} }} \left( {x,t} \right)$ and $V_{{2_{4} }} \left( {x,t} \right)$ are illustrated by the form of periodic soliton wave solutions designed for the ideals $\alpha = 3, \beta = 2, \zeta = 1, \varepsilon = 1, \mu = 1$ in the interval $0 < x < 50$ and $0 < t < 20$, $0 < x < 90$ and $0 < t < 70$ respectively are symbolized by Fig. 5 and 6. The profile of Fig. 7 in solution $V_{{2_{5} }} \left( {x,t} \right)$ shows the wave shape namely compacton for the values $\alpha = 3, \;\beta = 2, \;\zeta = 1, \;\varepsilon = 1, \;\mu = 1$ in the interval $0 < x < 5.5$ and $0 < t < 12.5$. A compacton is a special type of soliton that has a compact spatial profile, meaning it is limited to a finite region. Remarkably, compacton maintain their coherent shape after collisions, due to their soliton-like properties. The solution $V_{{2_{6} }} \left( {x,t} \right)$ for $\alpha = 3, \beta = 2, \zeta = 1, \varepsilon = 1, \mu = 1$ which indicates singular anti-bell-shaped soliton wave solution has cropped here in Fig. 8 within the range $0 < x < 0.1$, $0 < t < 2$. In addition, the solution $V_{{2_{7} }} \left( {x,t} \right)$ illustrates in Fig. 9 depicts the compacton wave solution for $\alpha = 3, \beta = 2, \zeta = 1, \varepsilon = 1, \mu = 1$ in the duration $0 < x < 70$, $0 < t < 75$. Finally, the solution $V_{{2_{8} }} \left( {x,t} \right)$ in Fig. 10 indicates the singular bell-shaped wave solution within the specified interval $0 < x, t < 1$. The graphs with similar shapes for the other two solutions ${V}_{{2}_{9}}\left(x,t\right)$ and ${V}_{{2}_{10}}\left(x,t\right)$ are omitted to maintain simplicity.

## Results comparison

In this part, we compare our achieved results with other existing results in the literature with the other methods. Those suggested equations presented above were examined by earlier researchers using a variety of methods. The improved Bernoulli sub-equation function approach with beta-derivative has been used to solve the above-mentioned equations and we attain several remarkable outcomes that are both more wide-ranging and relevant than those of the previous researchers. A comparison between existing solutions and our obtained solutions is presented in Tables 1 and 2.

**Table 1:** A comparison between the solutions obtained by Ege and Misirli^28^ and the solutions obtained by our study for the space–time fractional nonlinear Klein–Gordon equation using the IBSEF method.

Obtained solutions	Ege and Misirli^28^
If $\alpha \ne \beta , a = 1,b = 2, p_{0} = 1, q_{0} = 2,\tau = 1,$ then (4.1.9) solution becomes: $V_{{1_{1} }} \left( {x,t} \right) = \frac{{\sqrt 2 + \frac{2\beta \sqrt 2 }{{\alpha \left( { - \frac{\beta }{\alpha } + \frac{1}{{e^{{2\alpha \left( {\frac{m}{\gamma }\left( {x + \frac{1}{{{{\Gamma \gamma }}}}} \right)^{\gamma } + \frac{k}{\gamma }\left( {t + \frac{1}{{{{\Gamma \gamma }}}}} \right)^{\gamma } } \right)}} }}} \right)}}}}{2}.$ If $\alpha \ne \beta , a = 1,b = 2, p_{0} = 1, q_{0} = 2, q_{1} = 1,\tau = 1,$ then (4.1.15) solution becomes: $V_{{1_{5} }} \left( {x,t} \right) = \frac{{\sqrt 2 + \frac{\sqrt 2 }{{2\left( {\sqrt { - \frac{\beta }{\alpha } + \frac{1}{{e^{{2\alpha \left( {\frac{m}{\gamma }\left( {x + \frac{1}{{{{\Gamma \gamma }}}}} \right)^{\gamma } + \frac{k}{\gamma }\left( {t + \frac{1}{{{{\Gamma \gamma }}}}} \right)^{\gamma } } \right)}} }}} } \right)}}}}{{2 + \frac{1}{{\left( {\sqrt { - \frac{\beta }{\alpha } + \frac{1}{{e^{{2\alpha \left( {\frac{m}{\gamma }\left( {x + \frac{1}{{{{\Gamma \gamma }}}}} \right)^{\gamma } + \frac{k}{\gamma }\left( {t + \frac{1}{{{{\Gamma \gamma }}}}} \right)^{\gamma } } \right)}} }}} } \right)}}}}.$	If we put $b=c=1,$ in solution (39) then solution ${u}_{5}(x, t)$ becomes: $u_{5} \left( {x, t} \right) = \left( { - 1 + 2\frac{1}{{1 + e^{{\omega x + \frac{{\left( {\sqrt { - 2 + \omega^{2} } } \right)t^{\alpha } }}{{{\Gamma }\left( {1 + \alpha } \right)}}}} }}} \right).$ If we put $b = c = 1,$ in solution (42) then solution $u_{5} \left( {x, t} \right)$ becomes: $u_{7} \left( {x, t} \right) = \left( { - 1 + 2\frac{1}{{1 + e^{{\omega x - \frac{{\left( {\sqrt { - 2 + \omega^{2} } } \right)t^{\alpha } }}{{{\Gamma }\left( {1 + \alpha } \right)}}}} }}} \right).$

**Table 2:** A comparison between the solutions obtained by Uddin et al.^34^ and the solutions obtained by our study for the space–time fractional modified regularized long-wave equation using IBSEF method.

Obtained solutions	Uddin et al.^34^
If $\alpha \ne \beta , p_{2} = 1, q_{0} = 2, \tau = 1,$ then (4.2.8) solution becomes: $V_{{2_{1} }} \left( {x,t} \right) = \frac{{\frac{\alpha }{2\beta } + \frac{1}{{\left( { - \frac{\beta }{\alpha } + \frac{1}{{e^{{2\alpha \left( {\frac{m}{\gamma }\left( {x + \frac{1}{{{{\Gamma \gamma }}}}} \right)^{\gamma } + \frac{k}{\gamma }\left( {t + \frac{1}{{{{\Gamma \gamma }}}}} \right)^{\gamma } } \right)}} }}} \right)}}}}{2}.$ If $\alpha \ne \beta , p_{0} = 1, q_{0} = 2, q_{1} = 1,\tau = 1,$ then (4.2.20) solution becomes: $V_{{2_{9} }} \left( {x,t} \right) =$ $\frac{{1 + \frac{1}{{2\left( {\sqrt { - \frac{\beta }{\alpha } + \frac{1}{{e^{{2\alpha \left( {\frac{m}{\gamma }\left( {x + \frac{1}{{{{\Gamma \gamma }}}}} \right)^{\gamma } + \frac{k}{\gamma }\left( {t + \frac{1}{{{{\Gamma \gamma }}}}} \right)^{\gamma } } \right)}} }}} } \right)}}}}{{1 + \frac{2}{{\sqrt { - \frac{\beta }{\alpha } + \frac{1}{{e^{{2\alpha \left( {\frac{m}{\gamma }\left( {x + \frac{1}{{{{\Gamma \gamma }}}}} \right)^{\gamma } + \frac{k}{\gamma }\left( {t + \frac{1}{{{{\Gamma \gamma }}}}} \right)^{\gamma } } \right)}} }}} }}}}.$	If we put $q_{0} = q_{1} = 1,$ in solution (4.15) then solution $u_{{2_{2} }} \left( {x, t} \right)$ becomes: $u_{{2_{2} }} \left( {x, t} \right) =$ $\frac{{\sqrt {\frac{3\upsilon \tau }{{\left( {\tau + 2} \right)}}} \exp \left[ {\left( {x - \frac{2\upsilon }{{\tau + 2}}\frac{{t^{\alpha } }}{\alpha }} \right)} \right] - \sqrt {\frac{3\upsilon \tau }{{\left( {\tau + 2} \right)}}} }}{{\exp \left[ {\left( {x - \frac{2\upsilon }{{\tau + 2}}\frac{{t^{\alpha } }}{\alpha }} \right)} \right] + 1}}.$ If we set $q_{0} = q_{1} = q_{ - 1} = 1,$ in solution (4.16) then solution $u_{{2_{3} }} \left( {x, t} \right)$ becomes: $u_{{2_{3} }} \left( {x, t} \right) = \frac{{1 + {\text{exp}}\left[ { - \left( {x - \frac{1}{3}\left( {3\upsilon - 1} \right)\frac{{t^{\alpha } }}{\alpha })} \right)} \right]}}{{1 + {\text{exp}}\left[ { - \left( {x - \frac{1}{3}\left( {3\upsilon - 1} \right)\frac{{t^{\alpha } }}{\alpha })} \right)} \right]}}.$ If we insert $q_{0} = q_{1} = q_{ - 1} = 1,$ in solution (4.17) then solution $u_{{2_{4} }} \left( {x, t} \right)$ becomes: $u_{{2_{4} }} \left( {x, t} \right) =$ $\frac{{\sqrt {\frac{3\upsilon \tau }{{\left( {\tau + 2} \right)}}} - \sqrt {\frac{3\upsilon \tau }{{\left( {\tau + 2} \right)}}} \exp \left[ { - \left( {x - \frac{2\upsilon }{{\tau + 2}}\frac{{t^{\alpha } }}{\alpha }} \right)} \right]}}{{1 + \exp \left[ { - \left( {x - \frac{2\upsilon }{{\tau + 2}}\frac{{t^{\alpha } }}{\alpha }} \right)} \right]}}.$

We observe that the derived solution ${V}_{{1}_{1}}\left(x,t\right)$ and ${V}_{{1}_{5}}\left(x,t\right)$ of space–time fractional NLKG equation are similar to Ege and Misirli^28^ solutions. The others solution ${V}_{{1}_{2}}\left(x,t\right)-{V}_{{1}_{4}}\left(x,t\right)$ and ${V}_{{1}_{6}}\left(x,t\right)$ attain in this study are completely new and innovative and also have not reported in the prior study.

It is noteworthy to see that the consequent solution $V_{{2_{1} }} \left( {x,t} \right)$ and $V_{{2_{9} }} \left( {x,t} \right)$ of space–time fractional mRLW equation are analogous to Uddin et al.^34^ solutions. The other solutions $V_{{2_{2} }} \left( {x,t} \right) - V_{{2_{8} }} \left( {x,t} \right)$ and $V_{{2_{10} }} \left( {x,t} \right)$ found in this study are completely fresh and cutting-edge and have not previously been reported.

It is important to note that the achieved solutions to the space–time fractional NLKG and mRLW equations are explain the relativistic electrons in atom theory, to understand the long-wave occupancy in the ocean, including tsunamis and tidal waves, the propagation of water waves in shallow water, accounting for dispersion and dissipation effects.

## Conclusion

In this research, the space–time fractional NLKG equation and mRLW equation have been studied to the potential IBSEF method to develop some fresh and more universal closed-form traveling wave solutions of those equations in the sense of beta-derivative. We achieved exponential function solutions and hyperbolic trigonometric function solutions for numerous values of free parameters including bell-shaped, kink-shaped, single soliton, periodic soliton, compaction, and anti-kink-shaped. These solutions are demonstrated by three types of diagrams namely three-dimensional plotline, plot of contour, and 2D plot by using Mathematica. The acquired solutions can describe different types of phenomena like plasma waves in complex media, the propagation of water waves in shallow water, accounting for dispersion and dissipation effects, long-wave occupancy dynamics in the ocean, including tsunamis and tidal waves, which are essential for coastal hazard assessment and maritime safety, and understanding the behavior of spinless ion in relativistic particles. The accuracy of all solutions obtained was confirmed by substituting the original equation in each case using the computational software Maple and found them correct. This approach not only yields identical solutions, but it also has the potential to identify novel solutions that have not been reported by other researchers. This study demonstrates that the proposed technique is a useful, effective, and compatible mathematical tool for solving a broad range of NLFPDEs that arise in various domains of mathematical physics and engineering. The implications are far-reaching, as the solutions acquired through this methodology not only open the doors to resolving immediate problems but also lay the foundation for comprehensive research exploration. We have studied the NLFPDEs having balance number one but there are lots of NLFPDEs whose balance number is two or more. The future research might get a gorgeous way in looking for solitary wave solutions to the other NLFPDEs by the suggested technique when the balance number is two or more.

## Data Availability

The datasets used and/or analyzed during the current study available from the corresponding author on reasonable request.
